# Functional and Interfacial Responses of Honeybee Pupa Water-Soluble Proteins to pH, Ionic Strength, and Sucrose

**DOI:** 10.3390/foods15050884

**Published:** 2026-03-05

**Authors:** Feiying Yu, Xuejiao Wang, Linyan Zhou, Chaofan Guo, Junjie Yi

**Affiliations:** 1 Faculty of Food Science and Engineering, Kunming University of Science and Technology, Kunming 650500, China; feiying_yu@163.com (F.Y.); wangxuejiao173@hotmail.com (X.W.); zhoulinyan916@hotmail.com (L.Z.); junjieyi@kust.edu.cn (J.Y.); 2 Yunnan Key Laboratory of Plateau Food Advanced Manufacturing, Kunming 650500, China

**Keywords:** honeybee pupa water-soluble proteins, pH, ionic strength, sucrose, functional properties, interface features

## Abstract

The interfacial and functional properties of water-soluble protein (WP) from honeybee pupa are highly sensitive to environmental conditions, which govern its applicability in food systems. This study investigated the effects of pH (3–11), ionic strength (0–1 M NaCl), and sucrose concentration (0–1 M) on the colloidal behavior, surface hydrophobicity, sulfydryl exposure, functional properties, and interfacial characteristics of WP. These findings provide valuable reference data for future processing of bee pupa protein. Acidic conditions (pH 3) resulted in a high surface hydrophobicity (H_0_). Conversely, alkaline conditions enhanced protein interfacial activity. Specifically, the foaming capacity (FC) increased significantly with pH, reaching 90.88% at pH 11, which was approximately 2.5 times higher than that at pH 5 (35.10%). Moderate ionic strength (≤0.05 M NaCl) exerted minimal effects on particle size, while high salt levels (≥0.5 M) promoted aggregation via salting-out, increasing H_0_ from 219.91 (0 M) to 459 (1 M). Sucrose had little impact on particle size but significantly altered system viscosity. Interfacial measurements confirmed that moderate ionic strength (0.05 M NaCl) combined with sucrose addition (0.05 M) improved protein spreadability, yielding low contact angles of 9.60° and 9.93°, respectively. From the perspective of oil–water interfacial tension, increased pH and moderate sucrose concentrations reduce interfacial tension, promoting protein adsorption, whereas high salt and high sugar concentrations inhibit surfactant activity. Functional property evaluations indicated that alkaline conditions enhance foaming and emulsifying activity. Under conditions near pH 5, both foam and emulsion stability were optimal (foam stability ~99.95%), while the emulsifying capacity (~64.83%) was achieved at pH 11. As ion concentration increases, EC decreases. Sucrose concentration has no significant effect on emulsifying properties. These findings provide a quantitative reference for the tailored processing of honeybee pupa protein as a functional ingredient in food systems.

## 1. Introduction

Edible insect proteins have attracted increasing attention as sustainable and high-quality alternative protein resources due to their favorable amino acid profiles, low environmental footprint, and functional versatility [1,2,3]. Moreover, insect fats are rich in polyunsaturated fatty acids, with a degree of unsaturation comparable to that found in fish and poultry fats. Furthermore, insects serve as an important source of micronutrients such as zinc and iron [4]. Edible insect protein shows potential in alternative product development. Incorporating male honey bees (drones) increases dietary fiber content in dough [5]. In baking applications, insect meal has been demonstrated to enhance the nutritional value and texture of muffins [6]. In meat emulsion systems, *Tenebrio molitor* L. protein can effectively partially replace porcine myosin [7], with its larval emulsion showing promising potential as a meat substitute in emulsion-type sausages [8].

Existing research indicates that insect protein exhibits excellent technical properties in food applications, such as stabilizing oil/water emulsions and forming colloidal structures with long-term stability [9,10]. Additionally, during optimized protein processing, its emulsifying and foaming properties can be enhanced through enzymatic hydrolysis or heat treatment [11]. Protein functionality in food matrices is largely governed by its structural conformation, colloidal stability, and interfacial adsorption behavior, which are highly sensitive to external factors such as pH, ionic strength, and the presence of low-molecular-weight solutes [12,13,14]. Huang et al. demonstrated in their study on Tenebrio Molitor protein that high-intensity ultrasonication under strongly alkaline conditions enhances the foaming properties of this protein [15]. The protein solubility, foaming properties, and emulsifying performance of black soldier fly (*Hermetia illucens*) larvae were significantly improved when extracted under extreme alkaline pH conditions [16]. Ionic strength further modulates protein–protein and protein–solvent interactions via electrostatic shielding, exerting complex and often non-monotonic effects on the aggregation, emulsification, and foaming properties of proteins [17,18]. Studies on the functional properties of *Acheta domesticus* [19] proteins in relation to ionic strength have already been conducted. Current research on the effects of ionic strength on insect proteins has primarily focused on the extraction process stage. Examples include *Tenebrio molitor* [20,21], *Acheta domesticus* [20], *Locusta migratoria* [20], and *Protaetia brevitarsis* [21]. Meanwhile, non-electrolytes such as sucrose primarily alter solution viscosity and hydration dynamics, stabilizing protein conformation without directly modifying electrostatic interactions [22,23]. In previous studies, sucrose increased the gelation temperature of whey protein and the final stiffness of the cooled gel [24]. The addition of sucrose enhances the foam stability of soy globulin aqueous solutions [25,26].

Among the edible insects, honeybee pupa protein is particularly promising, as it is rich in essential amino acids and bioactive components and is produced as a by-product of apiculture [2,27,28]. The large-scale cultivation of bee pupae offers advantages such as short cycles, high resource utilization, and environmental friendliness [2]. Bee pupa protein also demonstrates potential for functional development, such as emulsification [29], foaming [29], and gelling [30,31,32] properties, along with processing characteristics.

However, the functional and interfacial properties of honeybee pupa protein under varying environmental conditions, along with its structural characterization, have yet to be evaluated.

Therefore, the objective of this study is to systematically investigate the effects of pH, ionic strength, and sucrose concentration on the functional and interfacial properties of water-soluble proteins from honeybee pupa. In this work, the influence of these factors on the emulsifying, foaming, and interfacial properties of honeybee pupa proteins was examined first. Subsequently, structural characterization revealed the changes occurring in honeybee pupa proteins under these varying conditions, providing scientific rationale for their rational utilization in food formulations and processing.

## 2. Materials and Methods

### 2.1. Materials

Fresh honeybee pupa (*Apis mellifera ligustica*) were purchased from a local market in Kunming City, Yunnan Province, China. Drone pupa were collected at 19 to 21 days post-laying (counted from the date of egg laying). At this stage, the pupa were fully developed and exhibited an optimal milky white to pale yellow coloration, representing the prime harvesting period. Sodium hydroxide and hydrochloric acid were obtained from Damiao Chemical Reagent Factory (Tianjin, China), while 8-anilino-1-naphthalenesulfonic acid (ANS) was sourced from Hanwei Chemical Technology Co., Ltd. (Shanghai, China). The BCA Protein Assay Kit was obtained from Sangon Biotech Co., Ltd. (Shanghai, China). Glycine was sourced from Macklin Biochemical Technology Co., Ltd. (Shanghai, China). Potassium dihydrogen phosphate and disodium hydrogen phosphate were obtained from Damao Chemical Reagent Factory (Tianjin, China). All other related items were purchased from Kunming Meibo Biotechnology Co., Ltd. (Kunming, China). All reagents were of analytical grade.

### 2.2. Preparation of Water-Soluble Proteins from Honey Bee Pupa (WPs)

Following the method of Ning et al. [33], 100 g of fresh honeybee pupa were taken and mixed with 500 mL of 0.05 M phosphate buffer solution (pH 7.0, containing 0.1 mM PMSF). The mixture was stirred in an ice bath for 60 min, then centrifuged at 24,000× *g* for 20 min at 4 °C. This extraction was performed twice. After dialysis and freeze-drying, the water-soluble protein was obtained. Protein content was determined using the BCA method.

### 2.3. pH, Salt Treatment, and Sucrose Treatment of WP

Following the method described in Chang et al. [34] with modifications, each 5 wt% honeybee pupa protein fraction was dissolved in deionized water. After magnetic stirring for 1 h under ice bath and dark conditions, the solutions were stored overnight at 4 °C to promote extensive hydration and achieve diffusion equilibrium. For pH studies, the solution pH was adjusted to 3, 5, 7, 9, or 11 using 0.1 M NaOH or 0.1 M HCl, followed by continuous magnetic stirring for 1 h. With ionic strength treatment, aliquots of the above solutions were incubated at room temperature after adding NaCl (0, 0.01, 0.05, 0.5, and 1 M). For sucrose treatment, aliquots of the above solutions were incubated at room temperature after adding sucrose (0, 0.01, 0.05, 0.5, and 1 M). Freshly prepared solutions were used for each measurement.

### 2.4. Characterization of Processed WP

#### 2.4.1. Particle Size Distribution

The particle size distribution of the samples was determined via a laser diffraction particle size analyzer (Mastersizer 3000, Malvern Panalytical, Malvern, Worcestershire, UK).

#### 2.4.2. Surface Hydrophobicity

Surface hydrophobicity (H_0_) was determined using the fluorescent probe 8-aniline-1-naphthalenesulfonate (ANS). Protein samples were sequentially diluted in 0.01, 0.05, 0.25, 0.75, and 3.75 mg/mL. A total of 1 mL of each diluted concentration were taken, add 12 μL of 8.0 mM ANS solution were added, vortex mixed, and incubated in the dark for 3 min to allow ANS to bind fully with the protein. Subsequently, the fluorescence intensity was measured at an excitation wavelength of 390 nm and an emission wavelength of 470 nm, with both excitation and emission slit widths set to 5 nm. To eliminate autofluorescence background, each measured value was corrected by subtracting the fluorescence intensity of the protein solution at the corresponding concentration without ANS. The resulting value represented the relative fluorescence intensity (RFI) for that sample. Finally, a linear fit was performed with protein concentration on the *x*-axis and relative fluorescence intensity on the *y*-axis. The initial slope of the resulting curve was determined as the surface hydrophobicity index H_0_ [30]. Note that H_0_ has no physical dimensions.

#### 2.4.3. Free Sulfhydryl Groups and Total Sulfhydryl Groups

The Ellman method was employed to determine the thiol content of proteins. Tris–Gly buffer (0.086 M Tris, 0.09 M Gly, 0.004 M EDTA, pH 8.0) was prepared, followed by the addition of 8 M urea to obtain Tris–Gly–Urea buffer. Ellman’s reagent was prepared by dissolving 20 mg of DTNB in 5 mL of Tris–Gly buffer. For the assay, 2 mL of protein solution was mixed with 10 mL of Tris–Gly-Urea buffer. Then, 2 mL of the mixture was combined with 80 μL of Ellman’s reagent, thoroughly mixed, and allowed to react at room temperature for 15 min. Absorbance was measured at 412 nm [35]. The formula for calculating free and total sulfhydryl groups is as follows:

(1) $$SH(\mu mol/g\;protein)=73.53\times A_{412}\times D/C$$

in the equation, 73.53 = 10^6^/(1.36 × 10^4^), where 1.36 × 10^4^ is the molar extinction coefficient of Ellman’s reagent; *A*_412_ is the absorbance at 412 nm; *D* is the dilution factor; and *C* is the protein concentration (mg/mL).

#### 2.4.4. Static Contact Angle

Following the method described by Ran [36] with minor modifications, 1 mL of the treated protein suspension was deposited onto a microscope slide. After air-drying at room temperature, a 5 μL drop of deionized water was placed onto the dried particle surface. The droplet shape was recorded using an OCA20 instrument (Dataphysics Co., Ltd., Esslingen, Baden-Württemberg, Germany), and the contact angle was calculated. Each sample was measured in triplicate.

#### 2.4.5. Oil–Water Interface Tension

The interfacial tension between a treated bee pupa protein solution (2.5 wt%) and corn oil was determined using the suspension drop method. During the experiment, the two phases were placed in a syringe and a cuvette, respectively, forming a water phase suspension drop within the oil phase at 25 °C. Image capture was immediately performed. Results were calculated through image analysis combined with the Young–Laplace equation [37].

#### 2.4.6. Foaming Capacity (FC) and Foam Stability (FS)

The FC of honeybee pupa protein after different treatments was evaluated using a T25 Ultra-Turrax (IKA-Werke GmbH & Co., Breisgau-Hochschwarzwald, Baden-Württemberg, Germany) high-speed homogenizer test. Protein solutions (15 mL) were transferred to centrifuge tubes after recording their initial volume (*V*). Following 15 min of equilibration at 25 °C, the sample solution volume was recorded and homogenized at 18,000 rpm for 2 min. After agitation, the solution was promptly poured into a 50 mL graduated cylinder. The foam volume (*V_a_*) was immediately recorded, and monitoring continued for 30 min, with the foam volume at 30 min (*V*_30_) recorded [38].

(2) $$\mathrm{Foaming}\;\mathrm{Capacity}\;(\%)=\frac{V_{a}-V}{V}\times 100$$

(3) $$\mathrm{Foam}\;\mathrm{Stability}\;(\%)=\frac{V_{30}}{V_{a}}\times 100$$

where *V* is volume before stirring (mL), *V_a_* is volume after stirring (mL), and *V*_30_ is volume after resting.

### 2.5. Preparation of Emulsion

1 mL of corn oil was taken and mixed with 9 mL of WP solution subjected to different treatments. Subsequently, the mixture was homogenized at 15,000 rpm for 2 min to prepare the emulsion.

### 2.6. Determination of Emulsion Properties

#### 2.6.1. Emulsification Capacity (EC) and Emulsion Stability (ES)

The EC and ES were evaluated according to the method described by Fernández-Sánchez [39] with minor modifications. Corn oil was mixed with a 5% *w*/*v* honeybee pupa protein solution at a 1:9 (*v*/*v*) ratio. After high-speed shear emulsification at 10,000 rpm for 1 min, the total height of the emulsion and the height of the emulsified layer were measured immediately for the calculation of EC. The ES was assessed by recording changes in emulsified layer height after heating the emulsion at 80 °C for 30 min.

(4) $$\mathrm{EC}\;(\%)=\frac{H_{E}}{H_{T}}\;\times \;100\;$$

(5) $$\mathrm{ES}\;(\%)=\frac{H_{EH}}{H_{TH}}\;\times \;100$$

where *H_E_* is the height of the emulsified layer, *H_T_* is the height of the total content, *H_EH_* is the height of the emulsified layer after heating, and *H_TH_* is the height of the emulsified layer before heating.

#### 2.6.2. Particle Size Distribution

The droplet size distribution of emulsions was determined using a laser diffraction instrument (Mastersizer 3000, Malvern Panalytical, Malvern, Worcestershire, UK). Before measurement, emulsions were diluted with deionized water to avoid multiple scattering effects [40].

#### 2.6.3. Viscosity

The viscosity of the sample was measured using a rheometer at a shear rate of 0.1–100 s^−1^ and a temperature of 25 °C.

#### 2.6.4. Microstructure of Droplets

The microstructure of the emulsion was observed using a confocal microscope (Nikon AXR, Nikon Instruments Inc., Melville, NY, USA) equipped with a 40× objective. Green and Nile Red dyes were used to stain proteins and the oil phase, respectively, at volume ratios of 1:500 (*v*/*v*) for the dye-to-protein ratio and 1:5000 (*v*/*w*) for the dye-to-oil phase ratio. Subsequently, the stained emulsion samples were transferred onto concave microscope slides with glass coverslips. The excitation wavelengths for green light and Nile red were 533 nm and 488 nm, respectively.

### 2.7. Statistical Analysis

Unless otherwise stated, all measurements were performed in triplicate. Data are presented as mean ± standard deviation (SD). Statistical analyses were conducted using Origin 2021 software (OriginLab Corporation, Northampton, MA, USA). One-way analysis of variance (ANOVA) was performed to evaluate differences among groups, and *p*-values were reported. Significant differences were further assessed using Tukey’s post hoc test, with a significance threshold of *p* < 0.05.

## 3. Results

### 3.1. Processed WP Characterization

After extraction, the WP yield was 22.34%. The BCA assay detected a protein concentration of 332.38 mg/g in the WP.

#### 3.1.1. Particle Size Distribution

**Figure 1:**
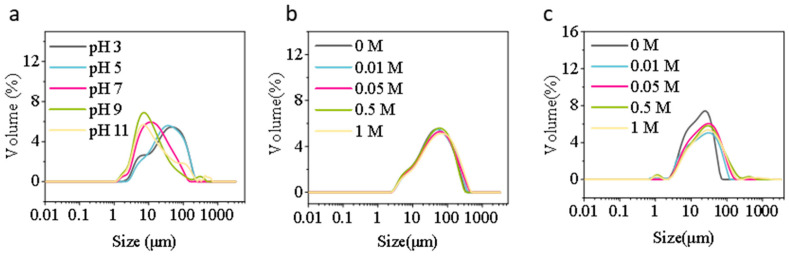
Particle size distribution (**a**–**c**) of honey bee pupa protein under (**a**) pH (3–11), (**b**) ionic strength (0–1 M), and (**c**) sucrose concentration (0–1 M).

First, the pH gradient (3, 5, 7, 9, 11) was examined (Figure 1a). When the pH ranges from 3 to 5, the particle size of the protein solution distribution peak is broad and exhibits a wide distribution range, indicating that the protein solution is prone to aggregation under acidic conditions. As pH increased to neutral to weakly alkaline conditions (pH 7–9), the distribution peak shifted toward smaller particle sizes of the protein solution (approximately below 10 μm) with a narrower peak shape, indicating enhanced charge repulsion and improved colloidal dispersion. At pH 11, the particle size of the protein solution distribution slightly increases, and the peak shifts slightly to the right. This may be due to the strong alkaline environment inducing partial unfolding or reorganization of the protein, leading to partial re-aggregation [41,42]. Second, observations at different ionic strengths (0, 0.01, 0.05, 0.5, 1 M) (Figure 1b) reveal minimal particle size variation in the protein solution under low-to-moderate salt conditions (0–0.05 M). However, at 0.5 M and 1 M, the particle size peak of the protein solution shifts markedly to the right, with larger particles appearing. This indicates that high ionic strength weakens electrostatic repulsion, promoting protein aggregation [43]. Previous studies have indicated that increased ionic strength enhances protein aggregation tendencies, thereby enlarging particle size [44]. Third, across sucrose concentration gradients (0, 0.01, 0.05, 0.5, 1 M) (Figure 1c), the particle size of the protein solution distribution showed minimal shift with concentration changes. Compared to Yu et al.’s study on egg white protein [45], the changes in WP were less pronounced, suggesting that the type of protein and processing methods may be contributing factors.

#### 3.1.2. Surface Hydrophobicity

**Figure 2:**
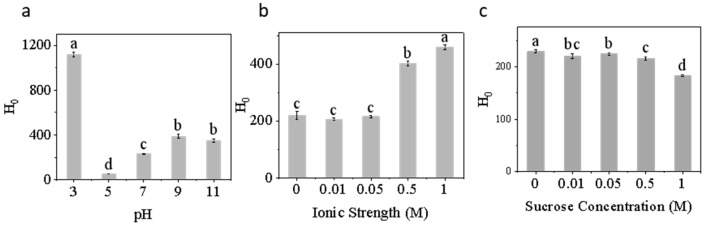
Surface hydrophobicity (H_0_) of honey bee pupa protein under (**a**) pH (3–11), (**b**) ionic strength (0–1 M), and (**c**) sucrose concentration (0–1 M). Note that H_0_ has no physical dimensions. Distinct lowercase letters denote statistically significant differences (*p* < 0.05).

As shown in Figure 2a, H_0_ exhibits strong pH dependence. The protein displays high hydrophobicity at pH 3 (≈1200), which is 4–6 times higher than under neutral or alkaline conditions. At pH 5, hydrophobicity decreases significantly. As pH increases, hydrophobicity rises. Under pH 3, the elevated hydrophobicity likely results from partial conformational destabilization, leading to exposure of hydrophobic domains. A similar phenomenon was reported by Mishyna et al., who demonstrated that acid-induced structural perturbation can increase the surface hydrophobicity of insect proteins [32]. As the pH continues to increase, H_0_ rises slightly again, likely due to the partial unfolding and exposure of hydrophobic residues under mild alkaline stress [46].

At low-salt levels (≤0.05 M), H_0_ remains relatively low and stable (Figure 2b). At ≥0.5 M, H_0_ markedly increased from 200 at 0 M to 450 at 1 M. This trend suggests that moderate ionic strength promotes electrostatic shielding, weakens repulsive forces between protein molecules, and exposes more hydrophobic regions [47]. However, the substantial increase at high ionic strengths suggests that salting-out effects and partial protein aggregation contribute to enhanced hydrophobicity [48].

Conversely, sucrose addition (0–1 M) exhibited an inverse relationship with H_0_ (Figure 2c). Hydrophobicity gradually decreased from ~220 (0 M) to ~180 (1 M). The reduction in H_0_ reflects sucrose’s exclusion from protein surface binding; instead, it is repelled to the protein exterior, promoting compact folding. This diminishes protein–protein interactions and limits the exposure of hydrophobic patches [49]. Sucrose molecules may form hydrogen bonds with polar residues and water, thereby maintaining compact tertiary structures and preventing exposure of hydrophobic surfaces [50].

#### 3.1.3. Free Sulfhydryl and Total Sulfhydryl Content

**Figure 3:**
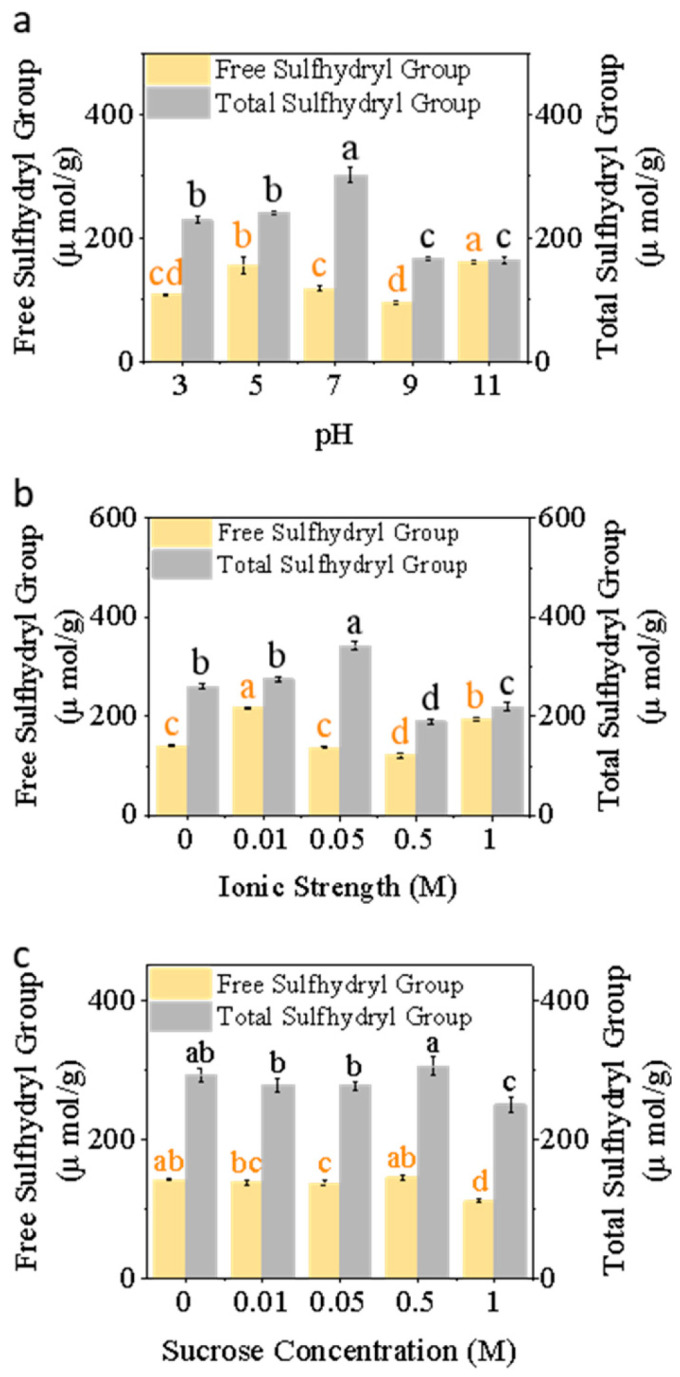
Free and total sulfhydryl groups of honeybee pupa protein under (**a**) pH (3–11), (**b**) ionic strength (0–1 M), and (**c**) sucrose concentration (0–1 M). Distinct lowercase letters denote statistically significant differences (*p* < 0.05).

As shown in Figure 3a, the exposure of total thiol groups (300 μmol/g) was highest at pH 7, while it was lowest at pH 9 and 11. This indicates that moderate pH favors a partially unfolded state, exposing thiol groups, whereas extreme acidic or alkaline conditions promote thiol oxidation or structural aggregation, leading to thiol loss or conversion to disulfide bridges [51]. This trend mirrors the pH-dependent behavior observed in microalgal proteins [52]. Free sulfhydryl content was highest at pH 11 compared to other conditions, but decreased between pH 5 and 9. This decline may result from partial protein unfolding, placing sulfhydryl groups in a highly reactive state susceptible to oxidation or disulfide bond formation [53]. Conversely, in extreme alkaline conditions, proteins unfold, exposing sulfhydryl groups and breaking disulfide bonds, leading to increased free sulfhydryl content [54].

As NaCl concentration increased from 0 to 1 M, both free and total sulfhydryl content exhibited nonlinear changes (Figure 3b). Free sulfhydryl levels increased from 150 μM/g at 0 M to 200 μM/g at 0.01 M, then decreased at higher ionic strengths (0.05–0.5 M) before rising again at 1 M. Total SH followed a similar trend. The initial increase indicates that moderate ionic strength disrupts protein interactions, enhancing protein unfolding and exposing buried thiol groups [55]. However, the subsequent decrease in free SH and total SH at high ionic strength suggests that disulfide bonds are oxidized or reorganized due to salting-out and aggregation, leading to tighter protein packing [56].

As sucrose concentration increased, free SH content remained relatively stable, while total SH peaked at 0.5 M (Figure 3c). Based on the observed reduction in free SH yet relatively high total SH at 1 M sucrose, we hypothesize that sucrose acts as a stabilizing osmotic regulator: by enhancing the protein hydration shell and reducing molecular mobility [57], it may suppress thiol exposure and inhibit the formation of new disulfide-linked aggregates [58].

#### 3.1.4. Static Contact Angle

**Figure 4:**
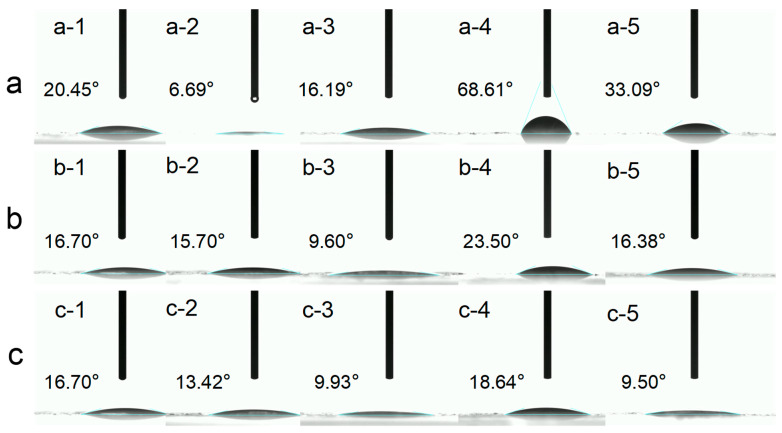
Interfacial properties of honeybee pupa protein (**a**) pH values (a-1: pH 3, a-2: pH 5, a-3: pH 7, a-4: pH 9, a-5: pH 11), (**b**) ionic strengths (b-1: 0 M, b-2: 0.01 M, b-3: 0.05 M, b-4: 0.5 M, b-5: 1 M), and (**c**) sucrose concentrations (c-1: 0 M, c-2: 0.01 M, c-3: 0.05 M, c-4: 0.5 M, c-5: 1 M).

The three-phase contact angle is a key parameter for quantifying protein wettability at the air–water interface. This method provides a useful and widely adopted indicator of protein surface wettability and conformational state [59]. It provides insights into the molecular orientation of proteins following their rearrangement at the interface. When proteins dry on a surface, they can rearrange, exposing different side chains (polar vs. nonpolar), which changes the hydrophilic/hydrophobic character and thus shifts the contact angle [60]. When the solution pH approached pH 5, the measured contact angle reached a minimum value (≈6.69°), indicating an almost complete wetting of the dried protein film by the liquid phase (Figure 4a). As the pH increased to 9, the contact angle significantly increased (approximately 68°), suggesting that the proteins, under alkaline conditions, carried more charges, resulting in enhanced electrostatic repulsion between molecules. This results in a less compact arrangement of the protein at the interface, thereby reducing wettability [61]. At pH 11, the contact angle slightly decreased to ~33°, potentially due to partial unfolding of the protein under highly alkaline conditions, exposing hydrophobic groups and enhancing interactions with the interface.

NaCl concentration exhibited a typical “salt effect” on protein interface behavior. At low-salt concentrations (0–0.01 M), the contact angle was approximately 16° (Figure 4b). As salt concentration increased to 0.05 M, the contact angle further decreased to 9.6°, indicating that moderate salt ions shield electrostatic repulsion between protein molecules, promoting aggregation and spreading at the interface while reducing surface hydrophobicity [62]. However, when salt concentration further increased to 0.5 M, the contact angle paradoxically increased (23.5°), suggesting that high salt levels induce protein conformational compression or aggregation, limiting their effective spreading at the interface. These results indicate that moderate ionic strength (~0.05 M) enhances protein interfacial adsorption performance, whereas excessively high salinity diminishes wettability.

As sucrose concentration increased (0–0.5 M), the contact angle exhibited an overall decreasing trend (Figure 4c). As a small-molecule polyhydroxy solute, sucrose stabilizes protein conformation and increases the flexibility of interfacial proteins by altering hydration and solution viscosity, facilitating their spreading at the interface. Additionally, sucrose may indirectly promote protein orientation and packing density at the interface by enhancing hydrogen bonding interactions between proteins and water molecules [63]. The results indicate that elevated sucrose concentrations favor improved wettability and reduced contact angles, consistent with their role in protecting protein structure and increasing solution viscosity.

#### 3.1.5. Oil–Water Interfacial Tension

**Figure 5:**
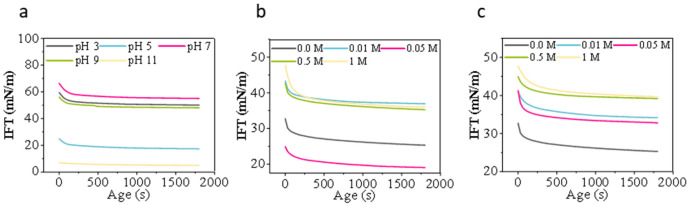
Oil–water interfacial tension (**a**) pH (3–11), (**b**) ionic strengths (0–1 M), and (**c**) sucrose concentrations (0–1 M).

At different pH conditions, IFT decreases significantly with increasing pH, consistent with the phenomenon of enhanced surface hydrophobicity in proteins at higher pH values. This indicates that the increased hydrophobic driving force promotes the adsorption of protein molecules at the oil–water interface, thereby enhancing the interfacial adsorption capacity of the proteins (Figure 5a). The highest IFT was observed under acidic conditions (pH 3), suggesting a more compact protein conformation with limited hydrophobic exposure [64]. In contrast, under alkaline conditions (pH 9–11), IFT markedly decreased as the protein partially unfolded. Exposure of hydrophobic residues promoted adsorption and spreading at the oil–water interface. Thus, alkaline conditions favored enhanced interfacial activity of bee pupa protein [59].

In Figure 5b, the 1.0 M sucrose concentration was higher than in the other groups. This indicates that increased ionic strength shields surface charges, weakens electrostatic repulsion, and promotes intermolecular aggregation, thereby reducing the protein’s diffusion rate and adsorption capacity at the interface [65].

As shown in Figure 5c, sucrose concentration exhibits a nonlinear trend on IFT, indicating complex effects of sucrose addition on proteins. At 0.05 M sucrose, oil–water surface tension decreases, suggesting that certain sucrose concentrations stabilize protein structures and promote interfacial migration. However, high sucrose concentrations (≥0.5 M) may limit protein diffusion and interfacial adsorption due to increased system viscosity [66].

#### 3.1.6. FC and FS

**Figure 6:**
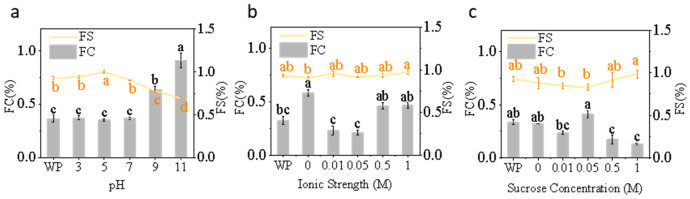
Foaming capacity and foam stability of honeybee pupa protein under (**a**) pH (3–11), (**b**) ionic strength (0–1 M), and (**c**) sucrose concentration (0–1 M). Distinct lowercase letters denote statistically significant differences (*p* < 0.05).

Under varying pH conditions, FC significantly increased from acidic to alkaline environments, peaking at pH 11—approximately 2.5 times higher than at pH 3 (Figure 6a). This pattern indicates that alkaline conditions enhance protein solubility and surface activity, promoting rapid unfolding and adsorption of molecules at the air–water interface [67]. This finding aligns with related studies on the foaming properties of chickpea protein [68]. This may be the reason for the increase in FC. In contrast, FS exhibits an opposite trend, showing maximum stability near pH 5. This may result from moderate protein–protein interactions near the isoelectric point promoting the formation of a dense interfacial film, which enhances foam stability [69]. Notably, under the influence of pH, the total sulfhydryl content decreases, exhibiting a similar trend to reduced foam stability. The reduction in available thiols at the interface limits the formation of disulfide cross-linking networks within the protein film at the gas–liquid interface, thereby weakening the strength of the interfacial film and leading to diminished foam stability [70].

As NaCl concentration increased (0–1 M), FC first decreased then increased, reaching its lowest value at 0.01–0.05 M and its highest at 0 M (Figure 6b). This biphasic behavior suggests that low ionic strength may shield electrostatic repulsion, leading to partial aggregation and reduced interfacial activity [71], while moderate ionic strength enhances molecular flexibility and promotes adsorption. This may be related to changes in FC. This trend is similar to the foaming properties observed in black cricket protein by Santiago et al. [72]. At different NaCl levels, FS remained relatively constant, suggesting that ionic effects primarily influence foam formation rather than stability [73]. The effect of NaCl (0.05–1 M) on FC follows the same trend as that on free sulfhydryl groups, as free sulfhydryl groups promote interfacial spreading and reduce surface tension.

Low concentrations of sucrose (0.01–0.05 M) slightly promoted protein adsorption at the interface, causing an increase in FC (Figure 6c). As sucrose concentration further increased, the rise in bulk viscosity restricted protein diffusion, suppressing foaming. However, FS exhibited minimal variation across all treatments, remaining above 0.6, suggesting sucrose primarily stabilizes the interfacial membrane rather than promoting foam generation [45].

### 3.2. Characteristics of WP Emulsions with Different Treatments

#### 3.2.1. EC and ES

**Figure 7:**
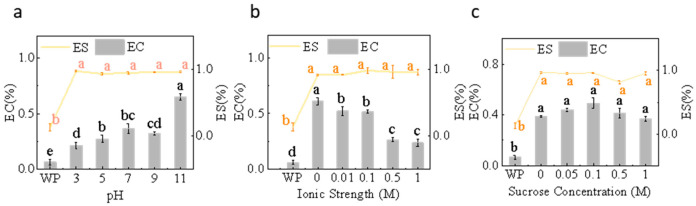
Emulsifying capacity and stability of honeybee pupa protein emulsions under (**a**) pH (3–11), (**b**) ionic strength (0–1 M), and (**c**) sucrose concentration (0–1 M). Distinct lowercase letters denote statistically significant differences (*p* < 0.05).

EC increases with rising pH, peaking at pH 11; ES remains high across the pH range of 5–11 (Figure 7a). pH alters protein surface charge and conformation. McCarthy et al. found that in environments far from alkaline conditions, proteins may carry higher net charges or undergo partial unfolding, accelerating their unfolding and adsorption rates at the oil–water interface, thereby enhancing EC [74]. Near the isoelectric point, although protein migration to the interface may decrease, moderate protein–protein interactions can form a denser interface film, thereby maintaining or enhancing ES [75]. This trend was also consistent with changes in IFT, where interfacial tension decreases, and interfacial adsorption increases in alkaline environments, manifesting as an increase in EC. Similar results were observed in the emulsification of *Rhynchophorus phoenicis* larvae, as reported by Fogang Mba et al. [76].

Under salt-free or low-salt conditions (0–0.05 M), EC is relatively high; as NaCl increases to medium-high concentrations (≥0.5 M), EC decreases significantly, whereas ES remains relatively stable with salt concentration, as shown in Figure 7b. Salt ions shield electrostatic repulsion by compressing the electric double layer [77]. Low to moderate salt concentrations may occasionally release some conformational constraints, temporarily improving interfacial adsorption [78]. However, as ionic strength continues to increase, salting-out and enhanced hydrophobic interactions promote protein aggregation or the formation of heterogeneous films at the interface [79], thereby reducing EC. Conversely, flexible structures formed by proteins at certain ionic strengths enhance interfacial membrane stability, leaving EC relatively unchanged or only slightly impaired [80].

When sucrose concentration increases from 0 to 1.0 M, EC shows a local increase at low concentrations (Figure 7c) followed by an overall decrease with a further concentration rise, while ES remains largely unchanged and maintains high levels under most treatments. It increases the viscosity of the continuous phase, limiting diffusion between protein molecules and oil droplets, leading to reduced EC at high concentrations. Furthermore, it stabilizes the protein conformation, such as inhibiting unfolding and aggregation [57]. As shown in Section 3.1.3, free sulfhydryl and total sulfhydryl content also correspond, potentially facilitating the formation of a denser interfacial film at medium-to-low sugar concentrations, manifested as localized EC increases and sustained ES. Previous reports on the effects of sugars on interfacial dynamics and stability in protein emulsification systems support this interpretation [81].

#### 3.2.2. Particle Size Distribution

**Figure 8:**
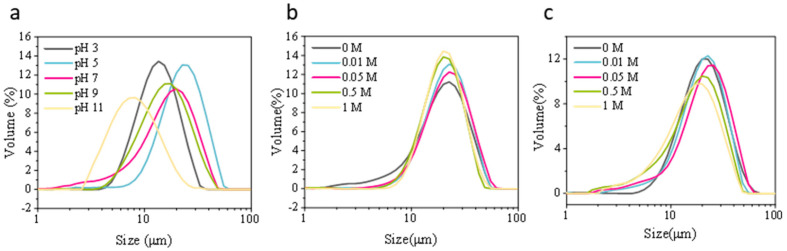
Particle size distribution of honeybee pupa protein emulsions under (**a**) pH (3–11), (**b**) ionic strength (0–1 M), and (**c**) sucrose concentration (0–1 M).

As pH increases from acidic to neutral and alkaline conditions, the emulsion particle size peak shifts toward smaller dimensions and exhibits a more concentrated profile (Figure 8a). At pH 3–5, the particle size peak is larger, and the distribution is broader. This reflects that under acidic conditions, proteins carry fewer charges and exhibit weaker electrostatic repulsion, making them prone to aggregation and thus forming larger emulsion oil droplets or clustered aggregates [82]. As pH approaches neutral to weakly alkaline, enhanced protein charge and stronger electrostatic repulsion inhibit large particle aggregation, resulting in a narrower size distribution with predominantly smaller particles [83]. This pH-dependent regulation of particle size aligns with previous studies on soybean/whey protein emulsions [82], indicating that pH alters protein conformation and adsorption behavior at the emulsion interface. At pH 11, further alkalinity causes negligible or slight increases in particle size, likely due to protein conformation rearrangement and re-exposure of hydrophobic regions triggering weak aggregation [84].

Under low-salt conditions (0–0.05 M), the particle size distribution showed little change (Figure 8b), indicating that moderate ionic strength can mitigate electrostatic repulsion between proteins without significantly compromising emulsion stability [85]. As NaCl concentration increased to 0.5–1.0 M, the particle size peak shifted slightly to the right toward larger dimensions. This shift primarily resulted from ion shielding effects and salt-induced protein–protein aggregation [78], leading to the formation of a less compact droplet coating layer at the interface.

Compared to pH and salt, sucrose concentration variations exerted a lesser influence on particle size. Within the 0–1.0 M sucrose gradient, particle size peaks remained within a similar range without significant broadening (Figure 8c). Previous studies have shown that water-soluble polysaccharides/sugars improve emulsion stability by restricting diffusion behavior within a certain concentration range but have no significant effect on particle size [86].

#### 3.2.3. Viscosity

**Figure 9:**
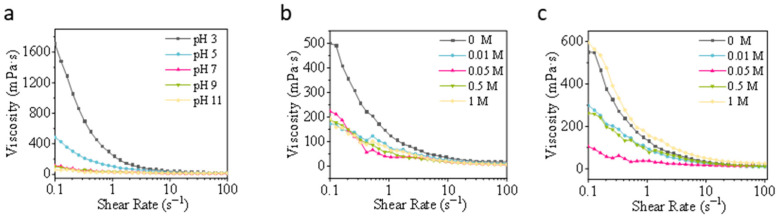
Apparent viscosity of honeybee pupa protein emulsions under (**a**) pH (3–11), (**b**) ionic strength (0–1 M), and (**c**) sucrose concentration (0–1 M).

As shown in Figure 9a, viscosity peaks at pH 3, with particularly pronounced differences in the low-shear range; it decreases sharply with increasing pH, decreasing sequentially at pH 5, 7, 9, and 11, with pH 7–11 approaching the low-viscosity level. Under acidic conditions, protein charges tend toward neutrality, weakening electrostatic repulsion and facilitating aggregation/reticular association, leading to the formation of large-sized/multiconnected aggregates [87]. This significantly increases low-shear viscosity. Far from the isoelectric point (neutral and alkaline conditions), proteins carry larger net charges, leading to enhanced electrostatic repulsion and improved dispersion. The system exhibits lower static viscosity [88]. This pattern aligns with particle size results, where acidic conditions yield larger particles.

Salt-free samples exhibit the highest viscosity under low-shear; viscosity decreases markedly upon adding small amounts of salt (0.01–0.05 M), then shows a recovery trend at medium-to-high salt concentrations (0.5–1 M), though remaining below the salt-free peak (Figure 9b). Low ionic strength shields long-range electrostatic repulsion, promoting partial unpacking or formation of more compact microaggregates in soluble components [71], thereby temporarily reducing viscosity. However, as ionic strength further increases, salting-out effects and electrostatic shielding drive stronger protein–protein interactions and reversible/irreversible aggregation, leading to a rebound in viscosity at low shear rates [89]. This non-monotonic variation corresponds to the aforementioned trend of increasing particle size with salt concentration.

Sucrose addition generally increases overall viscosity (0–1 M), with high sucrose concentrations (0.5–1 M) exhibiting significantly higher viscosity than the unsweetened system in the low-shear range (Figure 9c). Sucrose increases the viscosity of the solvent itself and the viscosity of the hydration layer. By altering the hydrophilic interactions between the hydration structure and proteins, it inhibits rapid large-scale protein motion, thereby maintaining higher viscosity in the dispersed system under low-shear conditions [90]. Low sucrose concentrations (0.05–0.05 M) exerted minimal or slightly reducing effects on viscosity.

#### 3.2.4. Microstructure of Liquid Droplets

**Figure 10:**
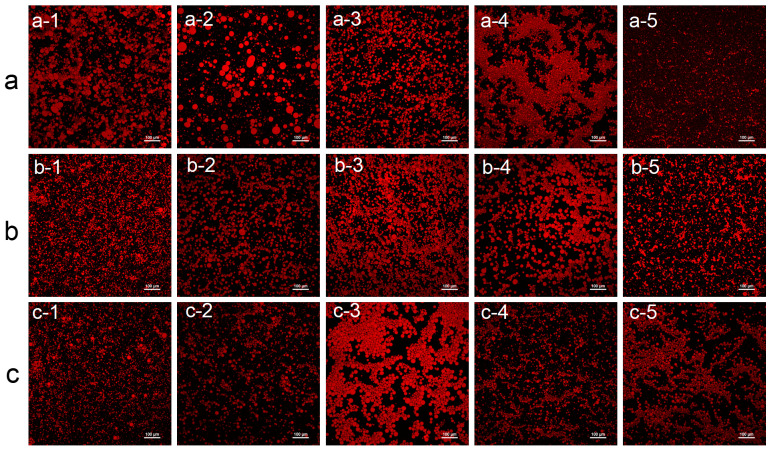
Emulsion droplet morphology (**a**) pH values (a-1: pH 3, a-2: pH 5, a-3: pH 7, a-4: pH 9, a-5: pH 11), (**b**) ionic strengths (b-1: 0 M, b-2: 0.01 M, b-3: 0.05 M, b-4: 0.5 M, b-5: 1 M), and (**c**) sucrose concentrations (c-1: 0 M, c-2: 0.01 M, c-3: 0.05 M, c-4: 0.5 M, c-5: 1 M). Note: scale bar: 100 μm.

In the pH gradient series, as pH increased from 3 to 11, the fluorescence distribution of emulsion particles exhibited systematic changes (Figure 10a). Specifically, particles showed higher aggregation at low pH conditions [91], while dispersion became more uniform at high pH, particularly exhibiting pronounced fluorescence halo effects at pH 11. Emulsion stability may increase under alkaline conditions.

In Figure 10b, the effect of NaCl was more pronounced: even low concentrations (0.01–0.05 M) caused slight particle aggregation; 0.5 M NaCl induced significant flocculation; and 1 M NaCl led to complete demulsification, forming extensive aggregated precipitates. This trend is similar to observations of globular proteins reported by Zhang et al. [89].

Sucrose concentration effects showed no significant impact on emulsion stability at low concentrations (0–0.05 M), with particles remaining uniformly dispersed (Figure 10c). However, phase separation began at 0.5 M, and at 1 M distinct fluorescent-enriched regions separated from non-fluorescent zones. This differs from the sodium caseinate emulsion formation results reported by Huck-Iriart et al. [92], potentially related to protein aggregation.

## 4. Conclusions

This study systematically elucidates the effects of pH, ionic strength, and sucrose concentration on the structure, interfacial properties, and functionality of water-soluble proteins from bee pupae. Results indicate that processing conditions play a decisive role in regulating protein conformation, colloidal stability, and interfacial behavior, thereby determining their functional performance in food systems. pH is the primary factor influencing protein structure and function. Under acidic conditions, reduced electrostatic repulsion promotes protein aggregation, leading to increased particle size, higher viscosity, enhanced surface hydrophobicity, and restricted interfacial activity. As pH gradually increases, enhanced protein charge and electrostatic repulsion improve dispersion, reduce particle size, and promote interfacial adsorption, thereby enhancing foaming capacity and emulsifying activity. Ionic strength exhibits typical salt dependence for honeybee pupae water-soluble proteins. Low ionic strength has limited effects on particle size and functional properties, whereas high ionic strength significantly weakens electrostatic repulsion and promotes aggregation through salting-out. This results in increased surface hydrophobicity, altered thiol kinetics, reduced emulsifying activity, and an impaired emulsion microstructure. Notably, moderate ionic strength improves interfacial spreading and reduces the contact angle, indicating enhanced interfacial adsorption efficiency within the optimal salt concentration range. As a non-electrolyte, sucrose primarily influences protein behavior by altering hydration and solution viscosity. Throughout the sucrose concentration range, emulsion particle size and droplet distribution remained largely unchanged. However, sucrose effectively reduced surface hydrophobicity, stabilized sulfhydryl groups, increased system viscosity, and improved interfacial wettability. These effects suggest that sucrose stabilizes protein conformation, limits excessive unfolding, and enhances interfacial membrane integrity, thereby supporting foam and emulsion stability without significantly increasing formation efficiency. These findings reveal certain structure–function–interface correlations in the honeybee pupa protein under different processing conditions. This study provides a theoretical foundation for tailoring processing conditions to optimize the functional properties of bee pupa protein. The findings also support the potential application of honeybee pupa protein as a sustainable functional ingredient in emulsified and aerated foods, contributing to the diversification of alternative protein sources in future food systems.

Future studies should focus on elucidating the molecular-level adsorption kinetics and viscoelastic properties of interfacial protein films using advanced surface-sensitive techniques (e.g., QCM-D, AFM, ellipsometry). Particular attention should be given to hydration layer dynamics and their role in stabilizing interfacial networks. Integrating quantitative droplet size distribution, interfacial rheology, and long-term stability analysis. Additionally, experimentally optimizing extraction and processing conditions to balance conformational flexibility and structural integrity can serve as a reference for practical applications and large-scale production.

## Data Availability

The original contributions presented in the study are included in the article, further inquiries can be directed to the corresponding author.
